# 2793. Clinical Outcomes of Combination Therapy with Daptomycin and Ceftaroline for Treatment of Methicillin-Resistant Staphylococcus aureus Bloodstream Infection

**DOI:** 10.1093/ofid/ofad500.2404

**Published:** 2023-11-27

**Authors:** Ryan W Chapin, Christopher McCoy, Kendall Donohoe, Dimple Patel

**Affiliations:** Beth Israel Deaconess Medical Center, Boston, MA; Beth Israel Deaconess Medical Center, Boston, MA; Beth Israel Deaconess Medical Center, Boston, MA; University of Cincinnati Medical Center, Cincinnati, Ohio

## Abstract

**Background:**

IDSA MRSA Treatment Guidelines recommend vancomycin or daptomycin as first-line therapy for methicillin-resistant *Staphylococcus aureus* (MRSA) bloodstream infection (BSI). Limited data and few recommendations exist regarding combination treatment for MRSA BSI outside of prosthetic valve endocarditis (PVE). The alternative treatment option of combining daptomycin with ceftaroline for its synergistic mechanism possibly through β-lactam-induced reduction in cell wall cross-linking has been pursued. This regimen is less feasible for home administration and more information on when to de-escalate to monotherapy after blood culture clearance is needed. This study evaluated clinical outcomes for patients with MRSA BSI who received combination therapy with daptomycin and ceftaroline.

**Methods:**

This was a single-center, retrospective study evaluating hospitalized patients mostly with persistent MRSA BSI from 1/2019 to 7/2022 who received a combination of daptomycin and ceftaroline. Patients with a diagnosis of pneumonia, PVE or polymicrobial bacteremia were excluded. The primary endpoint assessed in-hospital mortality for patients on combination therapy. Secondary endpoints included readmission within 90 days, reoccurrence of bacteremia within 30 days, length of stay and adverse drug reactions during treatment.

**Results:**

Of 73 patients screened, 14 patients were included with the primary reason for exclusion being diagnosis of PVE. Notably, half of the patients had a history of IVDU, with the majority of patients (71%) being started on initial therapy with vancomycin. Blood culture clearance occurred within 3 days of starting the combination for 11 of 14 patients. Only 4/14 patients received combination therapy for > 14 days, in which one patient died on a total duration of 26 days.

**Figure:**
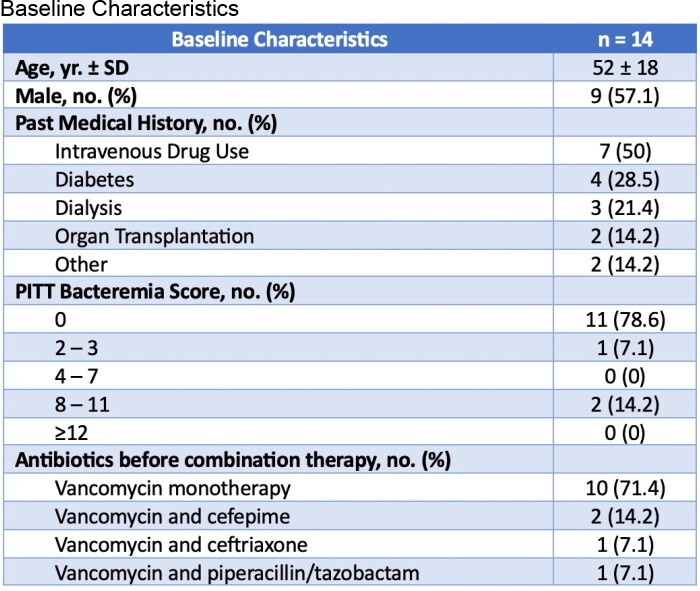

**Figure:**
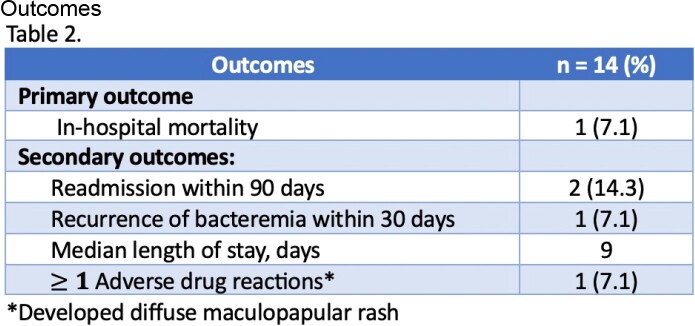

**Figure:**
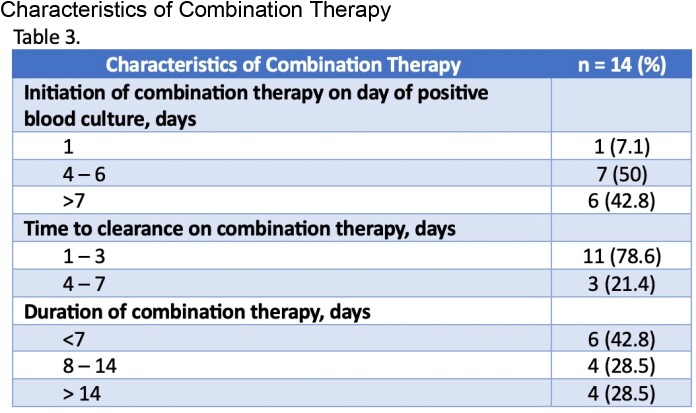

**Conclusion:**

This study demonstrated a variable duration of combination therapy for MRSA BSI in low-risk patients. The in-hospital mortality rate observed was low. All had microbiological cure. However, further exploration is needed to determine the optimal time for de-escalation after blood culture clearance. Future studies could contribute to creating stewardship efforts to de-escalate patients for MRSA BSI.

**Disclosures:**

**All Authors**: No reported disclosures

